# Portal Vein Thrombosis due to Prothrombin Gene Mutation following Sleeve Gastrectomy

**DOI:** 10.1155/2015/816914

**Published:** 2015-11-10

**Authors:** Murad Baba, Jordan Fakhoury, Amer Syed

**Affiliations:** ^1^Department of Internal Medicine, Jersey City Medical Center, 355 Grand Street, Jersey City, NJ 07302, USA; ^2^New York Institute of Technology College of Osteopathic Medicine, Old Westbury Northern Boulevard, Old Westbury, NY 11568, USA

## Abstract

*Introduction*. Portomesenteric thrombosis is increasingly recognized as a complication of laparoscopic sleeve gastrectomy (LSG). It often presents with abdominal pain. We present a mother and her son who both developed portal vein thrombosis (PVT) after LSG.* Case Description*. A 43-year-old woman presented complaining of sudden severe abdominal pain, two weeks after she had uncomplicated laparoscopic sleeve gastrectomy. CT scan of the abdomen and pelvis with IV contrast showed portal vein thrombosis and SMV thrombosis. Two weeks later her son had the same LSG for morbid obesity and presented with the same clinical picture. Thrombophilia workup showed heterozygous prothrombin gene mutation.* Conclusions*. A high index of suspicion is necessary to diagnose PVT; although rare, it can be potentially lethal. Anticoagulation therapy should be initiated immediately to limit the morbidities and improve the outcome. Patients with family history of thrombophilia should be investigated prior to any bariatric surgery and nonsurgical alternative treatments for morbid obesity should be strongly encouraged.

## 1. Introduction

The portal vein is formed by the splenic and superior mesenteric veins; it accounts for 75% of the blood supply to the liver (25% is accounted by the hepatic artery). Posterior to the neck of the pancreas, the portal vein originates from the union of the superior mesenteric and splenic veins. In the porta hepatis, the portal vein divides into the right and left branches that continue to their respective hepatic lobes, eventually emptying into hepatic sinusoids. Blood returning from the liver enters the inferior vena cava via the hepatic veins.

Portal vein thrombosis is a condition resulting from the formation of a blood clot in the extrahepatic portion of the portal vein. Local and systemic prothrombotic factors are both implicated in the development of PVTs. Infectious, inflammatory, and malignant conditions of the abdomen are the most commonly implicated local risk factors. Examples of systemic etiological factors include both congenital and acquired thrombophilic states. Recently, genetic polymorphisms such as Factor V Leiden and prothrombin 20210 gene mutation are being acknowledged as thrombogenic conditions resulting in splanchnic venous system clots [1].

According to the Centers for Disease Control and Prevention (CDC) more than 78 million Americans [2] (nearly one-third of all US adults) suffer from obesity. The American Society for Metabolic and Bariatric Surgery estimates that about 24 million have severe or morbid obesity [3]. Bariatric surgery has been shown to be the most effective and stable option of management for morbid obesity [4]. Surgery leads to significant weight loss and helps decrease the risks and improves or resolves more than 40 obesity-related diseases or conditions including certain cancers, type 2 diabetes, obstructive sleep apnea, and heart disease [5–7].

One common surgical procedure is the laparoscopic sleeve gastrectomy (LSG). LSG is a restrictive bariatric surgery procedure for high risk morbid obese patients, with lower incidence of complications and lower mortality rates in relation to the other bariatric operations. Beginning in the late 1990s, LSG was performed as the primary approach in bariatric surgeries before biliopancreatic diversion or Roux-en-Y gastric bypass [8]. This restrictive technique consisted of performing a longitudinal greater curvature gastrectomy starting proximal to the pylorus up to the gastroesophageal junction. The goal is to produce a restricted space with reduction in the size of the stomach to a 100 cc tube via greater curvature resection [9]. Although LSG is considered to be a safe procedure with fewer complications than other bariatric surgeries, there are still several complications to be considered [10]. An increasingly recognized yet rare complication of LSG is portomesenteric thrombosis. We report cases of postoperative PVT in a mother and her son who underwent LSG to treat morbid obesity.

## 2. Case Report

**Figure 1:**
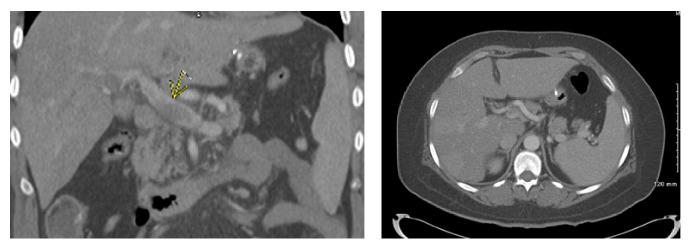
Contrast enhanced axial and coronal computed tomographic images of the abdomen. There is a lack of enhancement in the main portal vein (arrow) due to thrombosis.

A 43-year-old female patient, with PMH significant for morbid obesity, diabetes mellitus, GERD, and psoriasis, presented to the emergency room complaining of sharp severe abdominal pain that started two days ago in the epigastric area, got worse gradually, and spreads to the upper abdomen with radiation to the back. It was associated with nausea, without any other symptoms. Two weeks ago she had a laparoscopic sleeve gastrectomy and was sent home with noncomplicated postoperative recovery. On physical examination, her vital signs showed tachycardia of 111 bpm; abdomen examination revealed tenderness on palpation of the upper abdomen more in the epigastric area. Labs showed she is anemic with Hb 10.5 with normal kidney function, liver function, lactic acid, and coagulation profile. Further workup was done including a CT scan of the abdomen and pelvis with IV contrast that showed partial thrombosis of the main portal vein with complete thrombosis of the left portal vein and anterior branch of right portal vein as shown in Figure 1, in addition to superior mesenteric vein thrombosis.

She was admitted and started on therapeutic anticoagulation and her diet resumed gradually as tolerated. Her hospital course was smooth, and she was discharged on Rivaroxaban/Xarelto PO. Thrombophilia workup showed that she is negative for Factor 5 Leiden mutation but positive for one copy of the G20210A mutation (heterozygous) in the prothrombin/factor 2 gene.

Two weeks later her son, a 23-year-old male with PMH of DM and morbid obesity, had a laparoscopic sleeve gastrectomy two weeks ago and presented with sharp abdominal pain in the epigastric area with radiation to whole abdomen, physical exam showed abdominal tenderness on palpation and voluntary guarding, labs revealed AST of 115, ALT of 185, and CT scan of the abdomen with IV contrast showed evidence of portal vein thrombosis and SMV thrombosis, with hepatic artery collaterals draining the liver. His thrombophilia workup showed the same gene mutation as his mother.

## 3. Discussion

Portal vein thrombosis (PVT) is rare; usually it is associated with liver cirrhosis or prothrombotic disorders. It can be complete or partial. Patients with acute PVT may present clinically without symptoms or can have abdominal pain.

Symptoms and signs of PVT in the postoperative period are often nonspecific especially in obese patients. Abdominal pain, nausea, and vomiting are the usual chief complaints. Patients may also present with fever and elevated white blood cell count. To establish diagnosis, ultrasonography with Doppler imaging, computed tomography (CT), and magnetic resonance (MR) of the abdomen have replaced the customary invasive test of portal venography and superior mesenteric arteriography [1]. Acute PVT appears as a filling defect in the lumen of the portal vein upon imaging with CT scan with contrast [11]. CT scan can also be helpful in determining if any bowel ischemia or infarction has occurred.

Laparoscopic sleeve gastrectomy has significantly increased in utilization through the United States medical centers surpassing gastric bypass since 2013. Becoming the most commonly performed bariatric surgery, it makes up over 60% of all weight loss surgeries performed [12]. Given that its popularity has dramatically increased, physicians must be aware of the possible complications that may be associated with this procedure.

More common acute complications of LSG include hemorrhage, staple line leak, and abscess formation while more common chronic complications include stricture formation, nutritional deficiencies, and GERD [13]. Portal and mesenteric vein thrombosis are considered rare complications; however, they may be difficult to diagnose and indeed lead to dire consequences. Portomesenteric venous thrombosis accounts for 5% to 15% of all mesenteric ischemic events [14]. Clinical presentations range from incidental findings in completely asymptomatic patients to emergency bowel infarctions and ischemia [15].

Treatment for patients diagnosed with acute PVT (as with our patient) must be started immediately: underlying conditions should be recognized and interventional therapy and/or surgery must be considered. Physicians must then investigate any hypercoagulable states and patients are subjected to 3–6 months of anticoagulation. If it is determined that patients are indeed hypercoagulable, long-term anticoagulation must be considered with the goal of recanalization of the portal vein [16]; if a hypercoagulable state is ruled out, anticoagulation should be stopped in 3–6 months.

Portomesenteric vein thrombosis has been reported with several other laparoscopic surgical procedures including splenectomy, right hemicolectomy, and cholecystectomy [17, 18]. It is still uncertain what during laparoscopic procedures leads to the increased risk of thrombosis. It has been hypothesized that hemodynamic changes in pneumoperitoneum due to increased intra-abdominal pressure may play a role in thrombosis [19]. Furthermore, visceral vasoconstriction due to an increase of vasopressin intraoperatively, the retention of CO_2_ causing increased portal pressure, and patient positioning during surgery may all also affect portal blood flow. Several case reports [20–23] and studies [11, 24] have shown PVT occurring after laparoscopic sleeve gastrectomy. Most recently, a patient who underwent uncomplicated LSG presented two weeks after the surgery with epigastric pain was diagnosed with PVT, a case very similar to the one presented. However, the thrombophilia workup for that patient was unremarkable [20].

The risk of thromboembolism following bariatric surgery extends long after discharge from the hospital; this should raise the question of continuing thromboprophylaxis for several weeks postoperatively and it was suggested by some studies in the literature [25].

## 4. Conclusion 

Portal vein thrombosis represents an important clinical problem encountered by physicians. A high index of suspicion is necessary to diagnose this rare, but potentially lethal, complication. LSG is a safe procedure with low morbidity. Patients with family history of thrombophilia should be investigated prior to any bariatric surgery and nonsurgical alternative treatments for morbid obesity should be strongly encouraged.
